# Supplementary material for: Highly efficient UV/H2O2 technology for the removal of nifedipine antibiotics: Kinetics, co-existing anions and degradation pathways
Source: PLoS One. 2021 Oct 28;16(10):e0258483. doi: 10.1371/journal.pone.0258483 (PMC8553136; doi:10.1371/journal.pone.0258483)
Supplement: S1 Fig — (DOCX) [file pone.0258483.s001.docx]

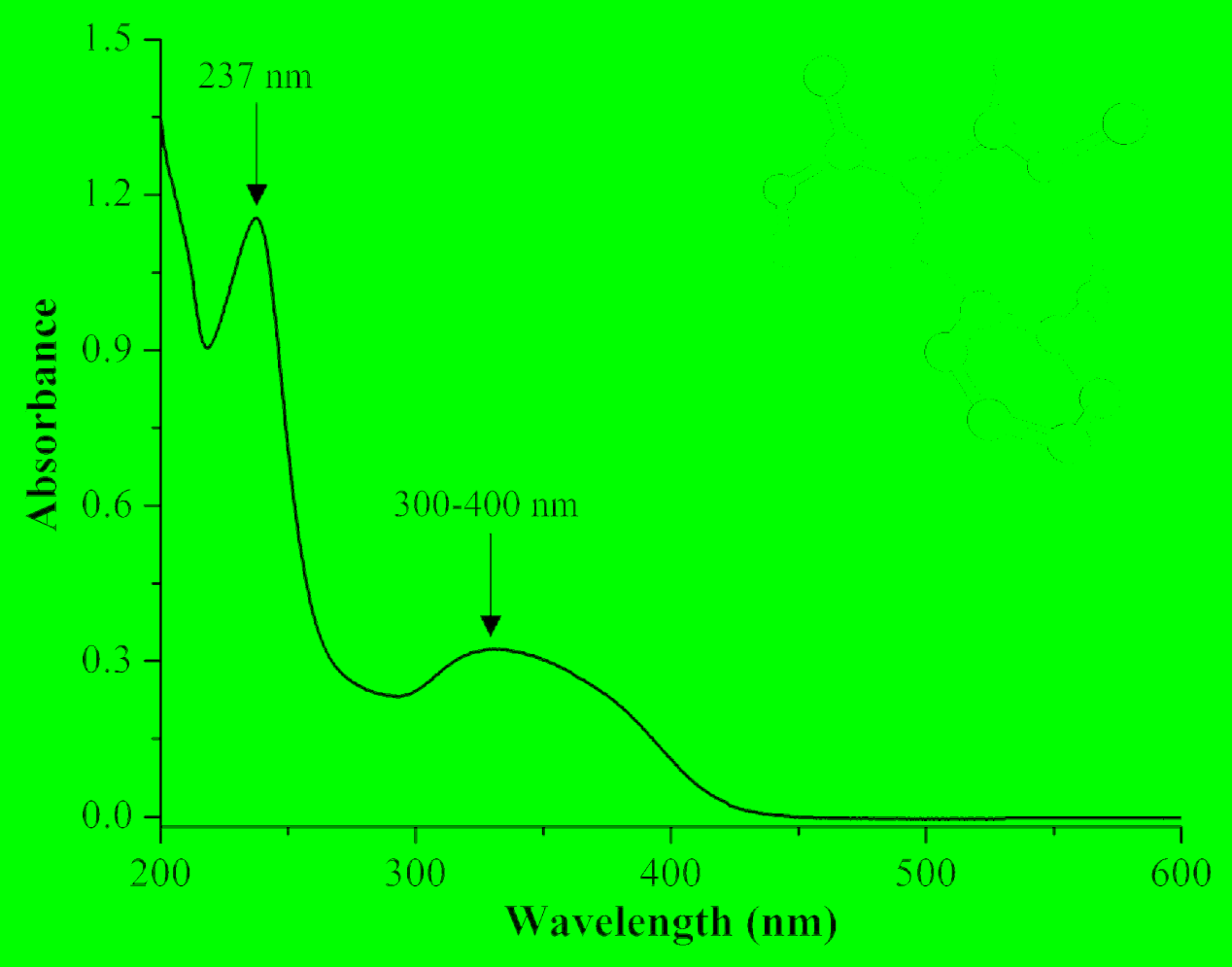


Fig. S1 UV-Vis absorption spectrum of NIF. Inset: Structural formula of NIF without H atoms (black - C atom; blue - N atom; red - O atom).

The characteristic absorption peak of NIF at 237 nm is derived from the π-π^*^ transition of C-C and C=C bonds^[1]^. The weak peak at 300-400 nm is attributed to the n-π^*^ transition of the aromatic π region^[2-3]^.
