# Supplementary material for: Highly efficient UV/H2O2 technology for the removal of nifedipine antibiotics: Kinetics, co-existing anions and degradation pathways
Source: PLoS One. 2021 Oct 28;16(10):e0258483. doi: 10.1371/journal.pone.0258483 (PMC8553136; doi:10.1371/journal.pone.0258483)
Supplement: S2 Fig — (DOCX) [file pone.0258483.s002.docx]

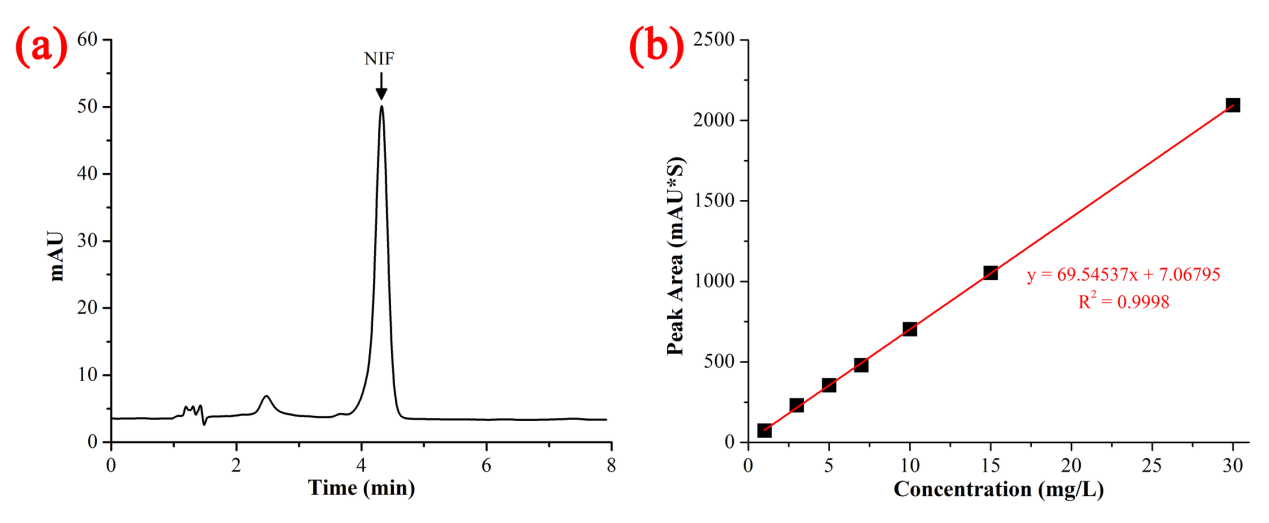


Fig. S2 Chromatography (a) and standard curve (b) of NIF.

The test wavelength of NIF in chromatography was 237 nm (Fig. S1). The appearance time of NIF in chromatography was 4.36 min^[4]^. The standard curve of NIF with 0-30 mg/L was y=69.54537x + 7.06795 with a good linear relation (R^2^ = 0.99981).
