# Supplementary material for: Highly efficient UV/H2O2 technology for the removal of nifedipine antibiotics: Kinetics, co-existing anions and degradation pathways
Source: PLoS One. 2021 Oct 28;16(10):e0258483. doi: 10.1371/journal.pone.0258483 (PMC8553136; doi:10.1371/journal.pone.0258483)
Supplement: S3 Fig — (DOCX) [file pone.0258483.s003.docx]

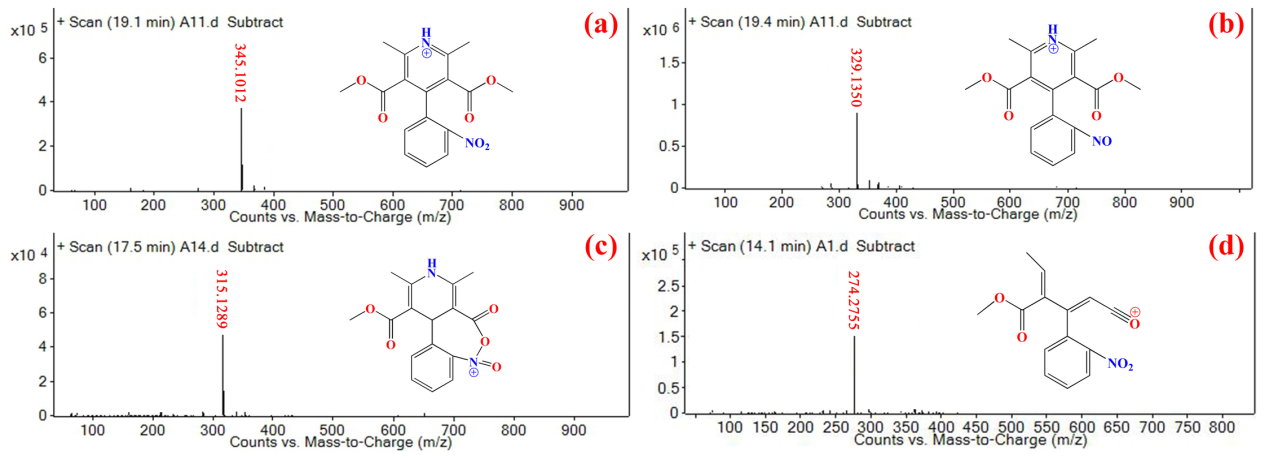


Fig. S3 Mass spectrum of intermediate products of NIF obtained *via* UV/H_2_O_2_.

Four intermediate products were present: P345 (*m/z* = 345, Fig. S3a), P329 (*m/z* = 329, Fig. S3b), P315 (*m/z* = 315, Fig. S3c) and P274 (*m/z* = 274, Fig. S3d)^[5]^.
