# Supplementary material for: Highly efficient UV/H2O2 technology for the removal of nifedipine antibiotics: Kinetics, co-existing anions and degradation pathways
Source: PLoS One. 2021 Oct 28;16(10):e0258483. doi: 10.1371/journal.pone.0258483 (PMC8553136; doi:10.1371/journal.pone.0258483)
Supplement: S4 Fig — (DOCX) [file pone.0258483.s004.docx]

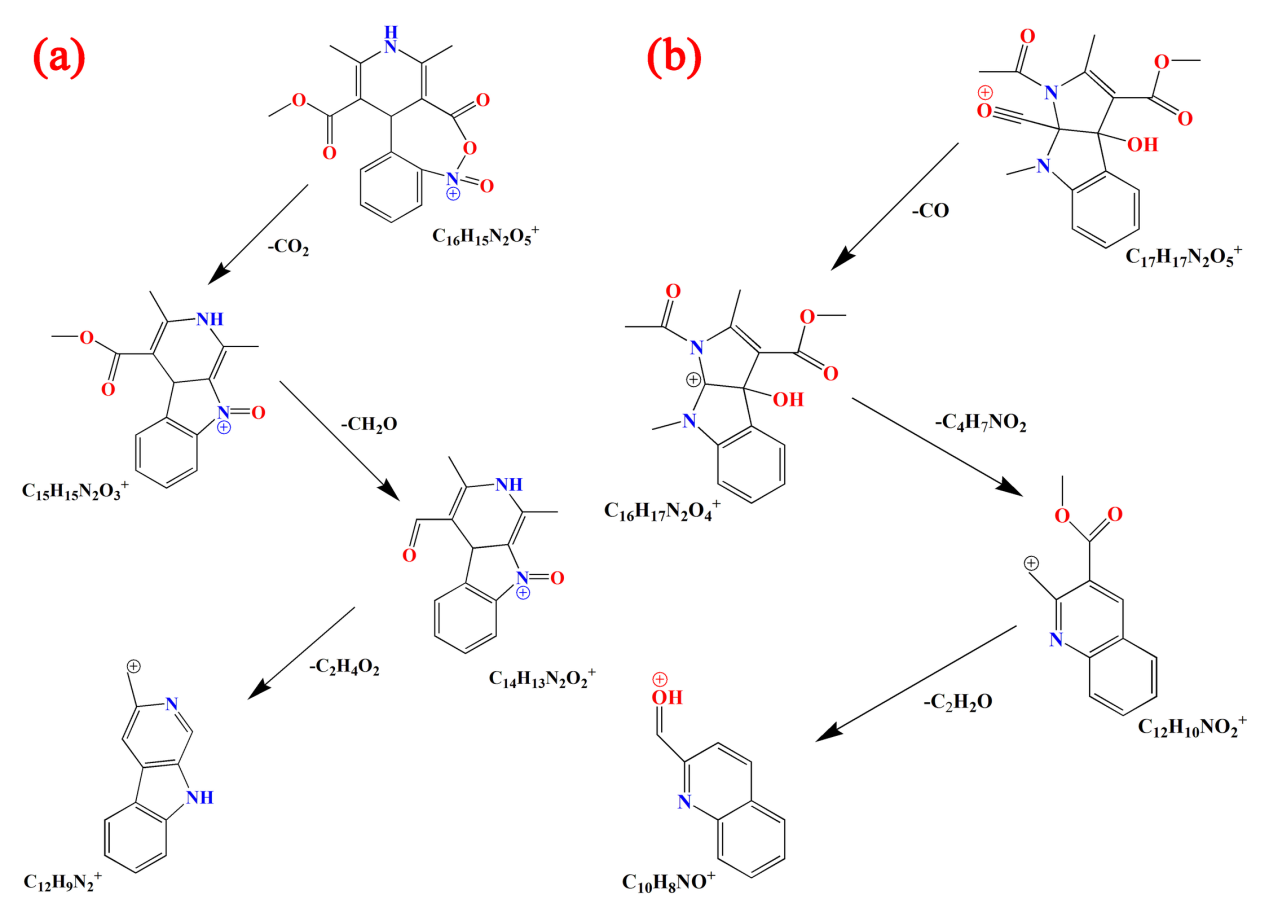


Fig. S4 Possible degradation pathways of P315 (a) and P329-2 (b) in the UV/H_2_O_2_ system.

As shown in Fig. S4a and Table S7, first, P315 (C_16_H_15_N_2_O_5_^+^, *m/z* = 315) lost CO_2_ to generate P271 (C_15_H_15_N_2_O_3_^+^, *m/z* = 271), then, P271 lost CH_2_O to generate P241 (C_14_H_13_N_2_O_2_^+^, *m/z* = 241), and finally, P241 lost C_2_H_4_O_2_ to generate P181 (C_12_H_9_N_2_^+^, *m/z* = 181)^[5]^.

As shown in Fig. S4b and Table S7, P329–2 (C_17_H_17_N_2_O_5_^+^, *m/z* = 329) first lost CO to generate P301 (C_16_H_17_N_2_O_4_^+^, *m/z* = 301). Next, P301 lost C_4_H_7_NO_2_ to generate P200 (C_12_H_10_NO_2_^+^, *m/z* = 200). Finally, P200 lost C_2_H_2_O to generate P158 (C_10_H_8_NO^+^, *m/z* = 158)^[5]^.
