# Supplementary material for: Highly efficient UV/H2O2 technology for the removal of nifedipine antibiotics: Kinetics, co-existing anions and degradation pathways
Source: PLoS One. 2021 Oct 28;16(10):e0258483. doi: 10.1371/journal.pone.0258483 (PMC8553136; doi:10.1371/journal.pone.0258483)
Supplement: S1 Table — Reaction conditions: NIF concentration = 5 mg/L, H2O2 dose = 0–1.04 mmol/L, pH = 7, T = 20 ℃ and reaction time = 5 min. (DOCX) [file pone.0258483.s005.docx]

Table S1. Effect of H_2_O_2_ dose on the degradation of NIF *via* UV/H_2_O_2_. Reaction conditions: NIF concentration = 5 mg/L, H_2_O_2_ dosage = 0-1.04 mmol/L, pH = 7, T = 20 ℃ and reaction time = 5 min.

| H_2_O_2_ dosages  mmol/L^-1^ | k’_app_  min^-1^ | Removal Rate  % | t_1/2_^a^  min | R^2^ |
| --- | --- | --- | --- | --- |
| 0 | 0.2560 | 72.81 | 2.8 | 0.99256 |
| 0.13 | 0.6752 | 95.97 | 1.5 | 0.98754 |
| 0.26 | 1.03947 | 99.31 | 1.0 | 0.98109 |
| 0.52 | 1.45569 | 99.94 | 0.4 | 0.99178 |
| 1.04 | 1.59404 | 99.95 | 0.6 | 0.98249 |
| a: t_1/2_ was half-life period (the reaction time with removal rate of NIF = 50%) | | | | |
