# Supplementary material for: Highly efficient UV/H2O2 technology for the removal of nifedipine antibiotics: Kinetics, co-existing anions and degradation pathways
Source: PLoS One. 2021 Oct 28;16(10):e0258483. doi: 10.1371/journal.pone.0258483 (PMC8553136; doi:10.1371/journal.pone.0258483)
Supplement: S2 Table — (DOCX) [file pone.0258483.s006.docx]

Table S2. Effect of initial pH on the degradation of NIF *via* UV/H_2_O_2_. Reaction conditions: NIF concentration = 5 mg/L, pH = 4-10, H_2_O_2_ dosage = 0.52 mmol/L, T = 20 ℃ and reaction time = 5 min.

| pH | k’_app_  min^-1^ | Removal Rate  % | t_1/2_  min | R^2^ |
| --- | --- | --- | --- | --- |
| 4 | 1.51175 | 99.94 | 0.7 | 0.99277 |
| 5 | 1.21831 | 99.77 | 0.8 | 0.99165 |
| 7 | 1.45569 | 99.94 | 0.4 | 0.99178 |
| 8 | 0.91269 | 98.69 | 0.9 | 0.97901 |
| 10 | 0.75217 | 97.54 | 1.1 | 0.99357 |
