# Supplementary material for: Highly efficient UV/H2O2 technology for the removal of nifedipine antibiotics: Kinetics, co-existing anions and degradation pathways
Source: PLoS One. 2021 Oct 28;16(10):e0258483. doi: 10.1371/journal.pone.0258483 (PMC8553136; doi:10.1371/journal.pone.0258483)
Supplement: S5 Table — (DOCX) [file pone.0258483.s009.docx]

Table S5. Effect of Cl^-^ on the degradation of NIF *via* UV/H_2_O_2_. Reaction conditions: NIF concentration = 5 mg/L, Cl^-^ concentration = 0-50 mg/L, pH = 7, H_2_O_2_ dosage = 0.52 mmol/L, T = 20 ℃ and reaction time = 5 min.

| Cl^-^ concentration  mg/L | k’_app_  min^-1^ | Removal Rate  % | t_1/2_  min | Inhibition Ratio^a^  % | R^2^ |
| --- | --- | --- | --- | --- | --- |
| 0 | 1.45569 | 99.94 | 0.4 |  | 0.99178 |
| 5 | 1.98350 | 100 | 0.3 | -36.25 | 0.98858 |
| 20 | 1.72666 | 99.98 | 0.3 | -18.61 | 0.98369 |
| 50 | 1.09588 | 99.57 | 0.9 | 24.71 | 0.99176 |
