# Supplementary material for: Highly efficient UV/H2O2 technology for the removal of nifedipine antibiotics: Kinetics, co-existing anions and degradation pathways
Source: PLoS One. 2021 Oct 28;16(10):e0258483. doi: 10.1371/journal.pone.0258483 (PMC8553136; doi:10.1371/journal.pone.0258483)
Supplement: S6 Table — (DOCX) [file pone.0258483.s010.docx]

Table S6. Effect of NO_3_^-^ on the degradation of NIF *via* UV/H_2_O_2_. Reaction conditions: NIF concentration = 5 mg/L, NO_3_^-^ concentration = 0-50 mg/L, pH = 7, H_2_O_2_ dosage = 0.52 mmol/L, T = 20 ℃ and reaction time = 5 min.

| NO_3_^-^ concentration  mg/L | k’_app_  min^-1^ | Removal Rate  % | t_1/2_  min | Inhibition Ratio  % | R^2^ |
| --- | --- | --- | --- | --- | --- |
| 0 | 1.45569 | 99.94 | 0.4 |  | 0.99178 |
| 5 | 1.03215 | 99.45 | 0.6 | 29.10 | 0.99813 |
| 20 | 1.29801 | 99.89 | 0.4 | 10.83 | 0.98198 |
| 50 | 1.55295 | 99.97 | 0.3 | -6.68 | 0.98583 |
