# Supplementary material for: Highly efficient UV/H2O2 technology for the removal of nifedipine antibiotics: Kinetics, co-existing anions and degradation pathways
Source: PLoS One. 2021 Oct 28;16(10):e0258483. doi: 10.1371/journal.pone.0258483 (PMC8553136; doi:10.1371/journal.pone.0258483)
Supplement: S7 Table — (DOCX) [file pone.0258483.s011.docx]

Table S7. Molecular weight, molecular formula, structural formula and *m/z* of intermediate products.

| Name | Molecular Weight | Molecular Formula | Structural Formula | *m/z* |
| --- | --- | --- | --- | --- |
| NIF | 347.35 | C_17_H_19_N_2_O_6_^+^ |  | 347.12 |
| P345 | 345.33 | C_17_H_17_N_2_O_6_^+^ |  | 345.11 |
| P274 | 274.25 | C_14_H_12_NO_5_^+^ |  | 274.07 |
| P329 | 329.33 | C_17_H_17_N_2_O_5_^+^ |  | 329.11 |
| P315 | 315.30 | C_16_H_15_N_2_O_5_^+^ |  | 315.10 |
| P271 | 271.30 | C_15_H_15_N_2_O_3_^+^ |  | 271.11 |
| P241 | 241.27 | C_14_H_13_N_2_O_2_^+^ |  | 241.10 |
| P181 | 181.22 | C_12_H_9_N_2_^+^ |  | 181.08 |
| P329-2 | 329.33 | C_17_H_17_N_2_O_5_^+^ |  | 329.11 |
| P301 | 301.32 | C_16_H_17_N_2_O_4_^+^ |  | 301.12 |
| P200 | 200.22 | C_12_H_10_NO_2_^+^ |  | 200.07 |
| P158 | 158.18 | C_10_H_8_NO^+^ |  | 158.06 |
