# Supplementary material for: Highly efficient UV/H2O2 technology for the removal of nifedipine antibiotics: Kinetics, co-existing anions and degradation pathways
Source: PLoS One. 2021 Oct 28;16(10):e0258483. doi: 10.1371/journal.pone.0258483 (PMC8553136; doi:10.1371/journal.pone.0258483)
Supplement: S8 Table — (DOCX) [file pone.0258483.s012.docx]

Table S8. Cost analysis of UV/H_2_O_2_ treatment.

| Materials | Prices | Price for treatment of 1 m^3^ wastewater |
| --- | --- | --- |
| 25 W UV lamp | $17.8 for 9000 h | $0.165 |
| H_2_O_2_ | $4.2 for 1 kg | $0.074 |
| Power | $0.1 for 1 kW·h | $0.208 |
| Total prices | $22.1 | $0.447 |
| Reference 47 | / | [$0.53, $0.85] |

**References**

[1] Lyu BW, Li HJ, Xue FF, Sai LM, Gui BJ, Qian DJ, Wang XY, Yang JH. Facile, gram-scale and eco-friendly synthesis of multi-color graphene quantum dots by thermal-driven advanced oxidation process. Chem Eng J. 2020. 388: 124285.

[2] Huo F, Liang WF, Tang YR, Zhang W, Liu XH, Pei DS, Wang HB, Jia WJ, Jia PP, Yang F. Full-color carbon dots with multiple red-emission tuning: on/off sensors, in vitro and in vivo multicolor bioimaging. J Mater Sci. 2019. 54: 6815-6825.

[3] Li SH, Jiang J, Yan YN, Wang P, Huang G, Kim NH, Lee JH, He DN. Red, green, and blue fluorescent folate-receptor-targeting carbon dots for cervical cancer cellular and tissue imaging. Mat Sci Eng C-Mater. 2018. 93: 1054-1063.

[4] Pietta P, Rava A, Biondi P. High-performance liquid chromatography of nifefipine, its metabolites and photochemical degradation products. J Chromatogr A. 1981. 210: 516-521.

[5] Handa T, Singh S, Singh IP. Characterization of a new degradation product of nifedipine formed on catalysis by atenolol: A typical case of alteration of degradation pathway of one drug by another. J Pharmaceut Biomed. 2014. 89: 6-17.
