# Supplementary material for: Highly efficient UV/H2O2 technology for the removal of nifedipine antibiotics: Kinetics, co-existing anions and degradation pathways
Source: PLoS One. 2021 Oct 28;16(10):e0258483. doi: 10.1371/journal.pone.0258483 (PMC8553136; doi:10.1371/journal.pone.0258483)
Supplement: Attachment — Submitted filename: reviewer.docx [file pone.0258483.s013.docx]

The manuscript “Highly efficient UV/H2O2 technology for the removal of nifedipine antibiotics”

Using UV/H2O2 technology is popular in removal organic contaminants. The author needs to mention the novel of this study in Introduction section.

Abstract:

- should write fully before using abbreviation, such as: NIF, ROS

- should add the co-existing anions effects

Introduction:

- Please add more removal techniques of NIF, and then find out the advantages and disadvantages of available techniques.

- The author needs to mention the novel of this study in Introduction section.

- Ref 10 & 12 were mentioned in Introduction section. In my opinion, the author reviewed the results of these researches clearly, but they did not release the advantages and disadvantages.

- The objectives of this study should be brief writing. Don’t need to describe the experiment, analysis and reaction pathway in Introduction section.

“Intermediate products analysis and degradation pathways were also obtained based on the Agilant 6520 series liquid chromatogram mass spectrometry (LC-Q-TOF-MS) system. There existed 11 kinds of intermediate products (P345, P329, P329-2, P315, P301, P274, P271, P241, P200, P181 and P158) and 2 degradation pathways (dehydrogenation reaction of NIF → P345 → P274 and dehydration reaction of NIF → P329 → P315)” 🡪 should be removed or rewrite.

- Agilant --> Agilent

Agilant 6520 series or Agilant 1260 series, please check it.

Materials and Methods:

- In Organics analysis section, please describe clearly the analysis programe

Results and discussion:

- From 3.1 to 3.6, the authors have just listed the results; please give some reasons to help the reader understand these effects.

- Could you give the comparison the effect between co-existing anions?

- Section 3.7 should be brief writing. If possible, move some discussion in this section to 3.1-3.6 sections.

- Author should add text to explain why “the degradation kinetics of NIF via UV/H2O2 system was linear decrease with SO42- concentration increasing.” “the degradation kinetics of NIF via UV/H2O2 system was linear decrease with CO32- concentration increasing” … instead of provide equations E3- E41.

- Please check SO42- in page 24 “The degradation rate of NIF decreases with increasing concentration of SO42- concentration, and the quenching mechanism of ROS via CO32- was shown as following equation 27 (E27) to equation 31 (E31)”
