# Supplementary material for: Highly efficient UV/H2O2 technology for the removal of nifedipine antibiotics: Kinetics, co-existing anions and degradation pathways
Source: PLoS One. 2021 Oct 28;16(10):e0258483. doi: 10.1371/journal.pone.0258483 (PMC8553136; doi:10.1371/journal.pone.0258483)
Supplement: Attachment — Submitted filename: PLOS.docx [file pone.0258483.s014.docx]

In the paper titled “Highly efficient UV/H2O2 technology for the removal of nifedipine antibiotics” authors have done systematic studies on removal of NIF in presence of UV and H2O2. The work is good but it needs some revision

1. The whole article has serious errors with English and the sentence drafting, it is highly urged to fix it.
2. Authors have used EPR studies, more experimental conditions like magnetic field, temperature etc need to be specified. This are very imp. parameters
3. Authors have repeated the data shown in different table in the manuscript as well while explanation, I think this is not comfortable and does not make any sense for the repetition of data. This needs to be removed. Authors should use values from different tables to explain their observations and restricts themselves by writing all the number in manuscript.
4. Authors should perform a study on the effect of NIF removal for different initial concentration of the NIF.
5. Authors have mentioned that pH of the water shows linear decrease with increase in pH value, however as far as I can see the from 4-7 the removal rate is almost constant. How to justify this?
