# Supplementary material for: Highly efficient UV/H2O2 technology for the removal of nifedipine antibiotics: Kinetics, co-existing anions and degradation pathways
Source: PLoS One. 2021 Oct 28;16(10):e0258483. doi: 10.1371/journal.pone.0258483 (PMC8553136; doi:10.1371/journal.pone.0258483)
Supplement: Attachment — Submitted filename: Response to Reviewers.doc [file pone.0258483.s015.doc]

**Response to Editor's comments:**

Thank you very much for your consideration of our manuscript entitled “**Highly efficient UV/H_2_O_2_ technology for the removal of nifedipine antibiotics: Kinetics, co-existing anions and degradation pathways**” (PONE-D-21-20580) to ***Plos One***.

We have read the editor’s comments and reviewers’ questions carefully, and made an attempt to respond as clearly as possible. In reference to the above recommendations, the responses are shown in “Response to 1^st^ reviewer”, “Response to 2^nd^ reviewer”, “Response to 3^rd^ reviewer” and “Response to 4^th^ reviewer”.

**Response to 1^st^ reviewer:**

***Question 1:*** *In the introduction critical comparison of the different methods for the removal of water pollutant needs to be included and the following references may be useful.*

*https://doi.org/10.1016/j.psep.2021.06.042*

*https://doi.org/10.1016/j.eti.2021.101581*

*https://doi.org/10.1016/j.jhazmat.2021.125960*

*https://doi.org/10.1007/s13399-020-01173-3*

*https://doi.org/10.1080/10643389.2020.1847949*

*https://doi.org/10.1016/j.envres.2021.111622*

**Answer.**

Thanks a lot. In **[Introduction]**, the critical comparison of the different methods for the removal of water pollutant was added. And the references ([13], [14], [15], [16], [17] and [18]) were cited.

Adsorption and advanced oxidation processes (AOPs, such as photocatalysis, Fenton, Fenton-like, photo-Fenton and catalytic ozonation) are the most promising wastewater treatment technologies for the removal of antibiotics from water environments and reduction of the resulting environmental risks because they are fast, efficient, low cost and convenient^[13-16]^. Many adsorbents have been employed for the eradication of antibiotics^[17]^. However, there are some drawbacks , such as incomplete removal, high energy requirements and the generation of toxic sludge and other waste products that entail further disposal^[18]^.

[13] Hasija V, Raizada P, Singh P, Verma N, Khan AAP, Singh A, Selvasembian R, Kim SY, Hussain CM, Nguyen VH, Le QV. Progress on the photocatalytic reduction of hexavalent Cr (VI) using engineered graphitic carbon nitride. Process Saf Environ. 2021; 152: 663-678.

[14] Soni V, Raizada P, Singh P, Cuong HN, Rangabhashiyam S, Saini A, Saini RV, Le QV, Nadda AK, Le TT, Nguyen VH. Sustainable and green trends in using plant extracts for the synthesis of biogenic metal nanoparticles toward environmental and pharmaceutical advances: A review. Environ Res. 2021; 202: 111622.

[15] Gunarathne V, Rajapaksha AU, Vithanage M, Alessi DS, Selvasembian R, Naushad M, You SM, Oleszczuk P, Ok YS. Hydrometallurgical processes for heavy metals recovery from industrial sludges. Crit Rev Env Sci Tec. 2020; 10.1080/10643389.2020.1847949.

[16] Hariharan A, Harini1 V, Sandhya S, Rangabhashiyam S. Waste *Musa acuminata* residue as a potential biosorbent for the removal of hexavalent chromium from synthetic wastewater. Biomass Convers Bior. 2020; 10.1007/s13399-020-01173-3.

[17] Rangabhashiyam S, Vijayaraghavan K, Jawad AH, Singh P, Singh P. Sustainable approach of batch and continuous biosorptive systems for praseodymium and thulium ions removal in mono and binary aqueous solutions. Environ Technol Inno. 2021; 23: 101581.

[18] Selvasembian R, Gwenzi W, Chaukura N , Mthembu S. Recent advances in the polyurethane-based adsorbents for the decontamination of hazardous wastewater pollutants. J Hazard Mater. 2021; 417: 125960.

***Question 2:*** *Include the photograph of the experimental setup or present the schematic representation.*

**Answer.**

The photograph of the experimental setup was added as Fig. 2 in **[Experimental setup]**.

**Fig. 2 Experimental setup of UV/H_2_O_2_.**

***Question 3:*** *Better present the effect of process parameters in figure representations.*
**Answer.**

The effect of H_2_O_2_ dose, initial pH and co-existing anions (SO_4_^2-^, CO_3_^2-^, Cl^-^ and NO_3_^-^) is presented as Fig.3, Fig.4, Fig.5, Fig.6, Fig.7 and Fig.8 in **[Results and discussion]**.

**Fig. 3 Effect of H_2_O_2_ dose on removal rate (a), kinetics constant (b) and linear fitting between kinetics constant and H_2_O_2_ dose on the degradation of NIF *via* UV/H_2_O_2_. The error bars represent the standard deviation (n = 3).**

**Fig. 4 Effect of initial pH on the removal rate (a), kinetics constant (b) and linear fitting between kinetics constant and initial pH on the degradation of NIF *via* UV/H_2_O_2_. The error bars represent the standard deviation (n = 3).**

**Fig. 5 Effect of SO_4_^2-^ on the removal rate (a), kinetics constant (b) and linear fitting between kinetics constant and concentration of SO_4_^2-^ on the degradation of NIF *via* UV/H_2_O_2_. The error bars represent the standard deviation (n = 3).**

**Fig. 6 Effect of CO_3_^2-^ on the removal rate (a), kinetics constant (b) and linear fitting between kinetics constant and concentration of CO_3_^2-^ on the degradation of NIF *via* UV/H_2_O_2_. The error bars represent the standard deviation (n = 3).**

**Fig. 7 Effect of CO_3_^2-^ on the removal rate (a), kinetics constant (b) and linear fitting between kinetics constant and concentration of CO_3_^2-^ on the degradation of NIF *via* UV/H_2_O_2_. The error bars represent the standard deviation (n = 3).**

**Fig. 8 Effect of NO_3_^-^ on the removal rate (a), kinetics constant (b) and linear fitting between kinetics constant and concentration of NO_3_^-^ on the degradation of NIF *via* UV/H_2_O_2_. The error bars represent the standard deviation (n = 3).**

***Question 4:*** *Perform the good number of cycles and present their results.*
**Answer.**

The UV/H_2_O_2_ system was not evaluated the recycleability due to the unrecoverable ability of H_2_O_2_. And we retrievaled the references of UV/H_2_O_2_ technology, the cyclic test of degradation of nifedipine *via* the UV/H_2_O_2_ method was not designed successfully.

***Question 5:*** *Report the cost analysis of the treatment process.*
**Answer.**

The cost analysis of UV/H_2_O_2_ system was added in **[Environmental significance]**.

In addition, the cost of the UV/H_2_O_2_ system was approximately $0.447 for 1 m^3^ wastewater (Table S8), which was lower than that of related systems (ranging from $0.53 to $0.85 for 1 m^3^ wastewater)^[47]^.

Table S8. Cost analysis of UV/H_2_O_2_ treatment.

| Materials | Prices | Price for treatment of 1 m^3^ wastewater |
| --- | --- | --- |
| 25 W UV lamp | $17.8 for 9000 h | $0.165 |
| H_2_O_2_ | $4.2 for 1 kg | $0.074 |
| Power | $0.1 for 1 kW·h | $0.208 |
| Total prices | $22.1 | $0.447 |
| Reference 47 | / | [$0.53, $0.85] |

***Question 6:*** *Error bar must be mentioned in all the experimental results presented.*
**Answer.**

The errer bar was added as Fig.3, Fig.4, Fig.5, Fig.6, Fig.7 and Fig.8 in **[Results and discussion]**.

See the answers of ***Question 3.***

**Response to 2^nd^ reviewer:**

***Question 7:*** *Why homogenous photocatalytic methods is preferred over methods are preferred over semiconductor photocatalyst are concerned. Please discuss in term of latest work.*

*https://doi.org/10.1016/j.cej.2019.123496*

*https://doi.org/10.1016/j.jphotochem.2019.01.015*

*https://doi.org/10.1016/j.arabjc.2018.12.001*

*doi:10.5004/dwt.2017.20831*

*https://doi.org/10.1016/j.jscs.2019.07.003*
**Answer.**

The comparison between homogenous photocatalyst and semiconductor photocatalyst was added in **[Introduction]**. And the references ([23], [24], [25], [26] and [27]) were cited.

However, the limitations of a wide bandgap, the rapid recombination rate of photogenerated electron-hole pairs, low solar light energy utilization efficiency, photocorrosion, and poor recyclability reduce the photocatalytic efficiency^[23-25]^. It is imperative to develop a novel Z-scheme system or heterojunction photocatalyst with broad photocatalytic applications^[26-27]^.

[23] Kumar A, Raizada P, Singh P, Saini RV, Saini AK, Hosseini-Bandegharaei A. Perspective and status of polymeric graphitic carbon nitride based Z-scheme photocatalytic systems for sustainable photocatalytic water purifcation. Chem Eng J. 2020; 391: 123496.

[24] Singh P, Shandilya P, Raizada P, Sudhaik A, Rahmani-Sani A, Hosseini-Bandegharaei A. Review on various strategies for enhancing photocatalytic activity of graphene based nanocomposites for water purification. Arab J Chem. 2020; 13: 3498-3520.

[25] Raizada P, Kumari J, Shandilya P, Singh P. Kinetics of photocatalytic mineralization of oxytetracycline and ampicillin using activated carbon supported ZnO/ZnWO_4_ nanocomposite in simulated wastewater. Desalin Water Treat. 2017; 79: 204-213.

[26] Raizada P, Sudhaik A, Singh P, Shandilya P, Gupta VK, Hosseini-Bandegharaei A, Agrawal S. Ag_3_PO_4_ modified phosphorus and sulphur co-doped graphitic carbon nitride as a direct Z-scheme photocatalyst for 2, 4-dimethyl phenol degradation. J Photoch Photobio A. 2019; 374: 22-35.

[27] Sonu, Dutta V, Sharma S, Raizada P, Hosseini-Bandegharaei A, Gupta VK, Singh P. Review on augmentation in photocatalytic activity of CoFe_2_O_4_ via heterojunction formation for photocatalysis of organic pollutants in water. J Saudi Chem Soc. 2019; 23: 1119-1136.

***Question 8:*** *The authors must mention the advantages photocatalysis over other treatment techniques. Please discuss with reference to pioneer work.*

*https://doi.org/10.1016/j.mtener.2020.100589.*

*https://doi.org/10.1039/D0TA08384D.*

*https://doi.org/10.1016/j.jece.2019.103272.4*

*https://doi.org/10.1016/j.seppur.2019.115692*
**Answer.**

The advantages of photocatalysis were added in **[Introduction]**. And the references ([19], [20], [21] and [22]) were cited.

Solar light-driven photocatalysis involves the photoinduced generation of holes (h^+^) in the valence band (VB) and electrons (e^-^) in the conduction band (CB) *via* light absorption by a semiconductor (TiO_2_, ZnO and CdS). Sequential interfacial charge transfers release various reactive oxygen species (ROS), such as superoxide, peroxide, and hydroxyl radicals, which participate in the degradation of organic and inorganic pollutants^[19-22]^.

[19] Kumar A, Raizada P, Hosseini-Bandegharaei A, Thakur VK, Nguyen VH, Singh P. C-, N-vacancies defect engineering polymeric carbon nitride meeting photocatalysis: Viewpoints and challenges. J Mater Chem A. 2020; 9: 111-153.

[20] Raizada P, Sudhaik A, Singh P, Hosseini-Bandegharaei A, Thakur P. Converting type II AgBr/VO into ternary Z scheme photocatalyst via coupling with phosphorus doped g-C_3_N_4_ for enhanced photocatalytic activity. Sep Purif Technol. 2019; 227: 115692.

[21] Hasija V, Sudhaik A, Raizada P, Hosseini-Bandegharaei A, Singh P. Carbon quantum dots supported AgI/ZnO/phosphorus doped graphitic carbon nitride as Z-scheme photocatalyst for efficient photodegradation of 2, 4-dinitrophenol. J Environ Chem Eng. 2019; 7: 103272.

[22] Patial S, Raizada P, Hasija V, Singh P, Thakur VK, Nguyen VH. Recent advances in photocatalytic multivariate metal organic frameworks-based nanostructures toward renewable energy and the removal of environmental pollutants. Mater Today Energy. 2021; 19: 100589.

***Question 9:*** *The graph must be drawn with good resolution*
**Answer.**

All the figures were drawn with good resolution of 300 dpi.

***Question 10:*** *How the temperature of 20 degree was maintained.*
**Answer.**

The temperature of 20 degree was maintained using thermostat water bath oscillator as Fig. 2 in **[Experimental setup]**.

**Fig. 2 Experimental setup of UV/H_2_O_2_.**

***Question 11:*** *The conclusion must be re-written in more conclusive way.*
**Answer.**

The conclusion was re-written as follows:

The degradation rate and degradation kinetics of NIF first increased and then remained constant as the H_2_O_2_ dose increased, and the quasi-percolation threshold was an H_2_O_2_ dose of 0.378 mmol/L. The effect of the initial pH, divalent anions (SO_4_^2-^ and CO_3_^2-^) and monovalent anions (Cl^-^ and NO_3_^-^) decreased linearly with increasing initial pH and co-existing anions (the R^2^ values of the initial pH, SO_4_^2-^, CO_3_^2-^ Cl^-^ and NO_3_^-^ were 0.6884, 0.9939, 0.8589, 0.5013 and 0.4514, respectively). ·OH was the primary key ROS, and ·O_2_^-^ was the secondary key ROS. There were 11 intermediate products (P345, P329, P329–2, P315, P301, P274, P271, P241, P200, P181 and P158) and 2 degradation pathways (dehydrogenation reaction of NIF → P345 → P274 and dehydration reaction of NIF → P329 → P315).

**Response to 3^rd^ reviewer:**

***Question 12:*** *Abstract:*

*- should write fully before using abbreviation, such as: NIF, ROS*

**Answer.**

The full name of NIF and ROS was added in **[Abstract]**.

This study investigates the degradation of nifedipine (NIF) by using a novel and highly efficient ultraviolet light combined with hydrogen peroxide (UV/H_2_O_2_).

The ·OH was the primary key reactive oxygen species (ROS) and ·O_2_^-^ was the secondary key ROS.

***Question 13:*** *Abstract:*

*- should add the co-existing anions effects*

**Answer.**

The co-existing anions effects were added in **[Abstract]**.

An increase in the initial pH and divalent anions (SO_4_^2-^ and CO_3_^2-^) resulted in a linear decrease of NIF (the R^2^ of the initial pH, SO_4_^2-^ and CO_3_^2-^ was 0.6884, 0.9939 and 0.8589, respectively). The effect of monovalent anions was complex; Cl^-^ and NO_3_^-^ had opposite effects: low Cl^-^ or high NO_3_^-^ promoted degradation, and high Cl^-^ or low NO_3_^-^ inhibited the degradation of NIF.

***Question 14:*** *Introduction:*

*- Please add more removal techniques of NIF, and then find out the advantages and disadvantages of available techniques.*

**Answer.**

The removal techniques of NIF were added in **[Introduction]**.

However, limited research has focused on the removal of NIF from the water environment *via* AOPs. Therefore, it is important to study the removal of NIF *via* AOPs for the treatment of medical wastewater.

Mojtaba Shamsipur et al. used a multivariate curve resolution method based on the combination of the Kubista approach and an iterative target transformation method by Gemperline to study the kinetics of NIF decomposition upon exposure to a 40 W lamp^[29]^. The results indicated that the photodecomposition kinetics of NIF are zero-order at the beginning of the reaction. However, when the reaction was more than 50% complete, the kinetics of the reaction changed to a first-order mechanism. The photo-degradation kinetics constants for the zero-order and first-order regions were (4.96±0.13)*10^-9^ M^-1^ s^-1^ and (6.22±0.10)*10^-5^ s^-1^, respectively. This was the first study on the degradation of NIF, but the low degradation rate (65%) and kinetics limited the application of NIF removal *via* a photo-degradation system.

***Question 15:*** *Introduction:*

*- The author needs to mention the novel of this study in Introduction section.*

**Answer.**

The novel of this study was mentioned in **[Introduction]**.

A novel method of UV light combined with hydrogen peroxide (UV/H_2_O_2_) is highly efficient, fast, and has a strong oxidizing ability; these advantages are attributed to the synergistic ability of UV light and H_2_O_2_ to generate ROS^[30]^. In our previous study, the degradation of norfloxacin by using UV/H_2_O_2_ was investigated^[31]^. The degradation rate and apparent first-order kinetics constant of norfloxacin *via* UV/H_2_O_2_ were 98.8% and 0.22248 min^-1^, respectively, and the norfloxacin concentration = 20 mg/L, the H_2_O_2_ dose =1.2 mmol/L, the pH=7, T= 20 °C and the reaction time = 20 min. The kinetics were low, and the formation mechanism of ROS was controversial, but it provided a novel research direction for the degradation of NIF *via* a UV/H_2_O_2_ system. Therefore, it should be noted that the degree of research to date on the degradation of NIF *via* UV/H_2_O_2_ oxidation processes is insufficient to thoroughly understand the fundamentals of ·OH generation, intermediate products and degradation pathways, which are important processes that must be considered in the design of wastewater treatment technology^[32]^. Furthermore, the effect of co-existing anions in the UV/H_2_O_2_ system may significantly affect the removal of NIF by competitively quenching with ROS^[33]^. Thus, it is still challenging to design a UV/H_2_O_2_ wastewater treatment technology with high efficiency.

***Question 16:*** *Introduction:*

*- Ref 10 & 12 were mentioned in Introduction section. In my opinion, the author reviewed the results of these researches clearly, but they did not release the advantages and disadvantages.*

**Answer.**

The advantages and disadvantages of reference 29 (Ref 10 of original manuscript) and 31(Ref 12 of original manuscript) were added in **[Introduction]**.

Mojtaba Shamsipur et al. used a multivariate curve resolution method based on the combination of the Kubista approach and an iterative target transformation method by Gemperline to study the kinetics of NIF decomposition upon exposure to a 40 W lamp^[29]^. The results indicated that the photodecomposition kinetics of NIF are zero-order at the beginning of the reaction. However, when the reaction was more than 50% complete, the kinetics of the reaction changed to a first-order mechanism. The photo-degradation kinetics constants for the zero-order and first-order regions were (4.96±0.13)*10^-9^ M^-1^ s^-1^ and (6.22±0.10)*10^-5^ s^-1^, respectively. This was the first study on the degradation of NIF, but the low degradation rate (65%) and kinetics limited the application of NIF removal *via* a photo-degradation system.

In our previous study, the degradation of norfloxacin by using UV/H_2_O_2_ was investigated^[31]^. The degradation rate and apparent first-order kinetics constant of norfloxacin *via* UV/H_2_O_2_ were 98.8% and 0.22248 min^-1^, respectively, and the norfloxacin concentration = 20 mg/L, the H_2_O_2_ dose =1.2 mmol/L, the pH=7, T= 20 °C and the reaction time = 20 min. The kinetics were low, and the formation mechanism of ROS was controversial, but it provided a novel research direction for the degradation of NIF *via* a UV/H_2_O_2_ system.

***Question 17*** *Introduction:*

*- The objectives of this study should be brief writing. Don’t need to describe the experiment, analysis and reaction pathway in Introduction section.*

**Answer.**

The objectives of this study were re-written in **[Introduction]**.

The aims of this study were to demonstrate the application of NIF degradation and to evaluate the performance and mechanism of UV/H_2_O_2_ AOPs. The specific objectives were (1) to assess the effect of the H_2_O_2_ dose, initial pH, and co-existing anions (SO_4_^2-^, CO_3_^2-^, Cl^-^ and NO_3_^-^) on the degradation of UV/H_2_O_2_, (2) to predict the key ROS of the UV/H_2_O_2_ method and (3) to propose the degradation pathway of NIF.

***Question 18*** *Introduction:*

*- “Intermediate products analysis and degradation pathways were also obtained based on the Agilant 6520 series liquid chromatogram mass spectrometry (LC-Q-TOF-MS) system. There existed 11 kinds of intermediate products (P345, P329, P329-2, P315, P301, P274, P271, P241, P200, P181 and P158) and 2 degradation pathways (dehydrogenation reaction of NIF → P345 → P274 and dehydration reaction of NIF → P329 → P315)” should be removed or rewrite.*

**Answer.**

The objectives of this study were re-written in **[Introduction]** and this sentences were removed.

***Question 19*** *Introduction:*

*- Agilant --> Agilent*

*- Agilant 6520 series or Agilant 1260 series, please check it.*

**Answer.**

“Agilant” was changed to “Agilent” in the manuscript.

In this study, the Agilent 1260 series liquid chromatogram mass spectrometry (LC-Q-TOF-MS) system (Agilent, USA) was used.

***Question 20*** *Materials and Methods:*

*- In Organics analysis section, please describe clearly the analysis programe.*

**Answer.**

NIF and its intermediate products in the UV/H_2_O_2_ degradation reaction solutions were analyzed by an Agilent 1260 series liquid chromatogram mass spectrometry (LC-Q-TOF-MS) system (Agilent, USA) with a C18 column (100 mm × 2.1 mm, 3.5 mm). The wavelength was 237 nm according to ultraviolet and visible spectrophotometry (Fig. S1). The mobile phase was methyl alcohol and deionized water at 63:35 (v/v). The drying gas of N_2_ was 8.0 mL/min, and the testing time was 30 min.

***Question 21*** *Results and discussion:*

*- From 3.1 to 3.6, the authors have just listed the results; please give some reasons to help the reader understand these effects.*

**Answer.**

The reasons for degradation of NIF was added in **[Results and discussion]**.

For **[3.1 Effect of H_2_O_2_ dose]** as following:

As shown in Fig. 3c, the effect of the H_2_O_2_ dose on the degradation of NIF *via* UV/H_2_O_2_ system had a dual nature. The degradation kinetics constant noticeably increased as the H_2_O_2_ dose increased and then remained constant at 1.5±0.1 min^-1^. When the H_2_O_2_ dose was < 0.52 mmol/L, the slope was 3.013 (min^-1^)/(mmol/L), but it decreased to 0.266 (min^-1^)/(mmol/L) when the H_2_O_2_ dose was > 0.52 mmol/L; hence, the quasi-percolation threshold (QPT) of the H_2_O_2_ dose was 0.378 mmol/L^[34]^. This trend was based on the generation and quenching of ·OH described by Equations 3 (E3) to 6 (E6)^[35]^:

(a) H_2_O_2_ dose < QPT:

H_2_O_2_ → 2 ·OH	(E3)

(b) H_2_O_2_ dose > QPT:

H_2_O_2_ + ·OH → ·HO_2_ + H_2_O	(E4)

·HO_2_ + ·OH → H_2_O + O_2_	(E5)

2 ·OH → H_2_O_2_	(E6)

**Fig. 3 Effect of H_2_O_2_ dose on removal rate (a), kinetics constant (b) and linear fitting between kinetics constant and H_2_O_2_ dose on the degradation of NIF *via* UV/H_2_O_2_. The error bars represent the standard deviation (n = 3).**

For **[3.2 Effect of initial pH]** as following:

As shown in Fig. 4c, the degradation kinetics of NIF *via* UV/H_2_O_2_ system exhibited a poor linear decrease as the pH increased (y = -0.1155x + 1.95533, R^2^ = 0.6884). The results indicated that acidic solution (pH = 4) was more favorable for degrading NIF than basic solution (pH = 10) under UV/H_2_O_2_. The possible reasons were in accord with the redox potential, generation rate of ROS and reaction rate between ROS and NIF. The inhibiting effect of the basic solution was due to the quenching reaction between OH^-^ and ·OH, which is shown in Equations 7 (E7) to 11 (E11)^[36]^:

H_2_O_2_ → HO_2_^-^ + H^+^	(E7)

H^+^ + OH^-^ → H_2_O	(E8)

H_2_O_2_ + HO_2_^-^ → H_2_O + OH^-^ + O_2_	(E9)

·OH + HO_2_^-^ → OH^-^ + ·HO_2_	(E10)

·OH + HO_2_^-^ → H_2_O + ·O_2_^-^	(E11)

**Fig. 4 Effect of initial pH on the removal rate (a), kinetics constant (b) and linear fitting between kinetics constant and initial pH on the degradation of NIF *via* UV/H_2_O_2_. The error bars represent the standard deviation (n = 3).**

For **[3.3 Effect of SO_4_^2-^]** as following:

As shown in Fig. 5c, the degradation kinetics of NIF *via* UV/H_2_O_2_ system exhibited a good linear decrease as the SO_4_^2-^ concentration increased (y = -0/0088x + 1.437, R^2^ = 0.9939). The inhibition effect was 5.08% (SO_4_^2-^ concentration = 5 mg/L) < 14.45% (SO_4_^2-^ concentration = 20 mg/L) < 31.20% (SO_4_^2-^ concentration = 50 mg/L), which was in keeping with the trend of the kinetics constants: 1.00154 min^-1^ (SO_4_^2-^ concentration = 50 mg/L) < 1.24540 min^-1^ (SO_4_^2-^ concentration = 20 mg/L) < 1.38175 min^-1^ (SO_4_^2-^ concentration = 5 mg/L). The degradation rate of NIF decreased with increasing SO_4_^2-^ concentration, and the quenching mechanism of ROS *via* SO_4_^2-^ is shown in Equations 12 (E12) to 17 (E17)^[37]^:

H^+^ + SO_4_^2-^ → HSO_4_^-^	(E12)

HSO_4_^-^ + ·OH → SO_4_·^-^ + H_2_O	(E13)

SO_4_·^-^ + H_2_O → H^+^ + SO_4_^2-^ + ·OH	(E14)

SO_4_·^-^ + H_2_O_2_ → H^+^ + SO_4_^2-^ + H_2_O·	(E15)

SO_4_·^-^ + H_2_O· → H^+^ + SO_4_^2-^ + O_2_	(E16)

SO_4_·^-^ + e^-^ → SO_4_^2-^	(E17)

**Fig. 5 Effect of SO_4_^2-^ on the removal rate (a), kinetics constant (b) and linear fitting between kinetics constant and concentration of SO_4_^2-^ on the degradation of NIF *via* UV/H_2_O_2_. The error bars represent the standard deviation (n = 3).**

For **[3.4 Effect of CO_3_^2-^]** as following:

As shown in Fig. 6c, the degradation kinetics of NIF *via* the UV/H_2_O_2_ system exhibited a good linear decrease as the CO_3_^2-^ concentration increased (y = -0.0072x + 1.3771, R^2^ = 0.8589). The inhibition trend was 11.00% (CO_3_^2-^ concentration = 5 mg/L) < 19.69% (CO_3_^2-^ concentration = 20 mg/L) < 28.25% (CO_3_^2-^ concentration = 50 mg/L), which was in keeping with that of kinetics constant: 1.04447 min^-1^ (CO_3_^2-^ concentration = 50 mg/L) < 1.16907 min^-1^ (CO_3_^2-^ concentration = 20 mg/L) < 1.29550 min^-1^ (CO_3_^2-^ concentration = 5 mg/L). The degradation rate of NIF decreased with increasing CO_3_^2-^ concentration, and the quenching mechanism of the ROS *via* CO_3_^2-^ is shown in Equations 18 (E18) to 22 (E22)^[38]^:

CO_3_^2-^ + H^+^ → HCO_3_^-^	(E18)

HCO_3_^-^ → CO_3_^2-^ + H^+^	(E19)

CO_3_^2-^ + ·OH → CO_3_^·-^ + OH^-^	(E20)

HCO_3_^-^ + ·OH → CO_3_^·-^ + H_2_O	(E21)

CO_3_^·-^ + H_2_O_2_ → HO_2_· + HCO_3_^-^	(E22)

**Fig. 6 Effect of CO_3_^2-^ on the removal rate (a), kinetics constant (b) and linear fitting between kinetics constant and concentration of CO_3_^2-^ on the degradation of NIF *via* UV/H_2_O_2_. The error bars represent the standard deviation (n = 3).**

For **[3.5 Effect of Cl^-^]** as following:

As shown in Fig. 7c, although the degradation kinetics of NIF *via* UV/H_2_O_2_ system decreased with increasing Cl^-^ concentration, the effect of Cl^-^ on the degradation of NIF had a dual nature: low Cl^-^ concentrations promoted the degradation of NIF, while high Cl^-^ concentrations inhibited the degradation of NIF. The degradation kinetics of NIF *via* UV/H_2_O_2_ system exhibited a poor linear decrease as the Cl^-^ concentration increased (y = -0.012x + 1.7897, R^2^ = 0.5013). The trend of inhibition was -36.25% (Cl^-^ concentration = 5 mg/L) < -18.61% (Cl^-^ concentration = 20 mg/L) < 24.71% (Cl^-^ concentration = 50 mg/L), which was in keeping with the trend of kinetics constant: 1.09588 min^-1^ (Cl^-^ concentration = 50 mg/L) < 1.72666 min^-1^ (Cl^-^ concentration = 20 mg/L) < 1.98350 min^-1^ (Cl^-^ concentration = 5 mg/L). The reaction mechanism between Cl^-^ and ·OH is shown in Equations 23 (E23) to 27 (E27)^[39]^:

Cl^-^ + ·OH → Cl· + OH^-^	(E23)

Cl· + Cl^-^ → ·Cl_2_^-^	(E24)

·Cl_2_^-^ + H_2_O_2_ → H^+^ + 2 Cl^-^ + H_2_O·	(E25)

·OH + Cl^-^ → ·HOCl^-^	(E26)

·HOCl^-^ + H^+^ → H_2_O + Cl^-^	(E27)

**Fig. 7 Effect of CO_3_^2-^ on the removal rate (a), kinetics constant (b) and linear fitting between kinetics constant and concentration of CO_3_^2-^ on the degradation of NIF *via* UV/H_2_O_2_. The error bars represent the standard deviation (n = 3).**

For **[3.6 Effect of NO_3_^-^]** as following:

As shown in Fig. 8c, NO_3_^-^ had the opposite effect on the degradation of NIF *via* the UV/H_2_O_2_ system compared to Cl^-^. The effect of NO_3_^-^ on the degradation of NIF had a dual nature: low NO_3_^-^ concentrations inhibited the degradation of NIF, but high NO_3_^-^ concentrations promoted the degradation of NIF. The degradation kinetics of NIF *via* the UV/H_2_O_2_ system showed a poor linear decrease with increasing NO_3_^-^ concentration (y = -0.005377x + 1.31881, R^2^ = 0.4514). The inhibition effect was -6.68% (NO_3_^-^ concentration = 50 mg/L) < 10.83% (NO_3_^-^ concentration = 20 mg/L) < 29.10% (NO_3_^-^ concentration = 5 mg/L), which was in keeping with the trend of the kinetics constant: 1.03215 min^-1^ (NO_3_^-^ concentration = 5 mg/L) < 1.29801 min^-1^ (NO_3_^-^ concentration = 20 mg/L) < 1.55295 min^-1^ (NO_3_^-^ concentration = 50 mg/L). The mechanism of the reaction between NO_3_^-^ and ·OH is shown in Equations 28 (E28) to 32 (E32)^[40]^:

NO_3_^-^ + *hv* → NO_2_^-^ + O	(E28)

NO_3_^-^ + *hv* → NO_2_· + O·^-^	(E29)

2 NO_2_· + H_2_O → NO_2_^-^ + NO ^-^ + 2 H^+^	(E30)

O + H_2_O → 2 ·OH	(E31)

O·^-^ + H_2_O → ·OH + OH^-^	(E32)

**Fig. 8 Effect of NO_3_^-^ on the removal rate (a), kinetics constant (b) and linear fitting between kinetics constant and concentration of NO_3_^-^ on the degradation of NIF *via* UV/H_2_O_2_. The error bars represent the standard deviation (n = 3).**

***Question 22*** *Results and discussion:*

*- Could you give the comparison the effect between co-existing anions?*

**Answer.**

The comparison of co-existing anions for degradation of NIF was added in **[Results and discussion]**.

In summary, the effect of co-existing anions was as follows: The divalent anions (SO_4_^2-^ and CO_3_^2-^) caused a good linear decrease with increasing initial pH and concentration of divalent anions (R^2^ of SO_4_^2-^ and CO_3_^2-^ was 0.9939 and 0.8589, respectively). The monovalent anions had a complex effect; Cl^-^ and NO_3_^-^ had opposite effects on NIF degradation: low Cl^-^ and high NO_3_^-^ promoted degradation, while high Cl^-^ and low NO_3_^-^ inhibited degradation. The degradation kinetics of NIF *via* the UV/H_2_O_2_ system showed a poor linear decrease with increasing Cl^-^/NO_3_^-^ concentration (R^2^ of Cl^-^ and NO_3_^-^ was 0.5013 and 0.4514, respectively).

***Question 23*** *Results and discussion:*

*- Section 3.7 should be brief writing. If possible, move some discussion in this section to 3.1-3.6 sections.*

**Answer.**

The **[3.7 Oxidation mechanism and degradation pathway of NIF via UV/H_2_O_2_]** was re-written. The related discussion in this section was moved to 3.1-3.6 sections as the answers of ***Question 21***.

In recent advances in UV/H_2_O_2_ systems, the degradation of organic pollutants has been due to the generation of ROS, especially ·OH and ·O_2_^-^. The oxidation mechanism of NIF degradation *via* the UV/H_2_O_2_ system was measured by ESR measurements, and the ESR spectra are shown in Fig. 9(a-b). The significant ·OH signal (Fig. 9a) showed four peaks at 321.8 mT (P_A_), 323.3 mT (P_B_), 324.8 mT (P_C_) and 326.3 mT (P_D_). The interspaces of P_A_-P_B_, P_B_-P_C_ and P_C_-P_D_ were constant of 1.5 mT, and the intensity ratio of P_A_, P_B_, P_C_ and P_D_ was 1:2:2:1^[41]^. The significant ·O_2_^-^ signal (Fig. 9b) showed four peaks at 322.1 mT (P’_A_), 323.2 mT (P’_B_), 324.4 mT (P’_C_) and 325.8 mT (P_D_). The interspaces of P’_A_-P’_B_, P’_B_-P’_C_ and P’_C_-P’_D_ were constant within the range of 1.0 mT from 1.5 mT, and the intensity ratio of P’_A_, P’_B_, P’_C_ and P’_D_ was 1:1:1:1^[42]^. However, the intensity of the ·OH signal was stronger than that of the ·O_2_^-^ signal, which means that ·OH was the primary key ROS and ·O_2_^-^ was the secondary key ROS. The oxidation mechanism of NIF degradation *via* the UV/H_2_O_2_ system is shown in Equations 33 (E33) to 41 (E41)^[43]^. The generation of ·OH mainly comes from the direct decomposition of H_2_O_2_ (E33), the generation of ·O_2_^-^ mainly comes from the indirect reaction between oxygen gas (dissolved oxygen and H_2_O_2_ decomposition) and electrons (E35 and E38), and NIF is degraded *via* the ROS (E41)^[44]^.

H_2_O_2_ + *hv* → 2 ·OH	(E33)

H_2_O_2_ + ·OH → ·HO_2_ + H_2_O	(E34)

·HO_2_ → ·O_2_^-^ + H^+^	(E35)

2 ·HO_2_ → H_2_O_2_ + O_2_	(E36)

·HO_2_ + ·OH → H_2_O + O_2_	(E37)

O_2_ + e^-^ → ·O_2_^-^	(E38)

2 ·O_2_^-^ → ^1^O_2_ + ^3^O_2_ + 2 e^-^	(E39)

2 ·OH → H_2_O_2_	(E40)

NIF + ROS → Intermediate Products → CO_2_ + H_2_O	(E41)

**Fig. 9 ESR measurements of ·OH (a) and ·O_2_^-^ (b).**

The identification of intermediate NIF products *via* UV/H_2_O_2_ was performed using an Agilent 1260 series liquid chromatogram mass spectrometry (LC-Q-TOF-MS) system. The reaction conditions were: NIF concentration = 5 mg/L, pH = 7, H_2_O_2_ dose = 0.52 mmol/L, T = 20 °C and reaction time = 30 min. Preliminary analyses were conducted to evaluate the intermediate products of NIF produced by the UV/H_2_O_2_ method, and four kinds of NIF degradation intermediate products were observed in the mass spectrum based on the mass-to-charge (*m/z*) ratio. Four intermediate products were present: P345 (*m/z* = 345), P329 (*m/z* = 329), P315 (*m/z* = 315) and P274 (*m/z* = 274). The structures of P345, P329, P315 and P274 are shown in Fig. S3a, Fig. S3b, Fig. S3c and Fig. S3d, respectively.

**Fig. 10 Chromatography of the intermediate products of NIF *via* UV/H_2_O_2_.**

As shown in Fig. 10(a-b), the peak areas of all the observed products first increased and then decreased within 30 min. The peak areas of P345 and P274 at different reaction times are shown in Fig. 10a, and the results indicated that the maximum peak area of P345 appeared at 5 min, the maximum peak area of P274 appeared at 20 min, and the peak intensity of P345 was stronger than that of P274. The first degradation pathway of NIF *via* the UV/H_2_O_2_ method was proposed as follows: protonated NIF (C_17_H_19_N_2_O_6_^+^, *m/z* = 347) first lost H_2_ to generate P345 (C_17_H_17_N_2_O_6_^+^, *m/z* = 345) *via* dehydrogenation reaction, and then P345 lost C_3_H_5_NO to generate P274 (C_14_H_12_NO_5_^+^, *m/z* = 274), as shown in Fig. 11a^[45-46]^. The peak areas of P329 and P315 at different reaction times are shown in Fig. 10b, and the results indicated that the maximum peak area of P329 appeared at 0.5 min, the maximum peak area of P315 appeared at 7 min, and the peak intensity of P329 was stronger than that of P315. The second degradation pathway of NIF *via* the UV/H_2_O_2_ method was proposed as follows: protonated NIF (C_17_H_19_N_2_O_6_^+^, *m/z* = 347) first lost H_2_O to generate P329 (C_17_H_17_N_2_O_5_^+^, *m/z* = 329) *via* dehydration reaction, and then P329 lost CH_2_ to generate P315 (C_16_H_15_N_2_O_5_^+^, *m/z* = 274), as shown in Fig. 11b^[45-46]^.

**Fig. 11 Degradation pathway of NIF *via* UV/H_2_O_2_ system.**

P315 was degraded with the attack of ROS. The intermediate products of P271 (*m/z* = 271), P241 (*m/z* = 241) and P181 (*m/z* = 181) are shown in Table S7, and its possible degradation pathway is shown in Fig. S4a. P315 first lost CO_2_ to generate P271 (C_15_H_15_N_2_O_3_^+^, *m/z* = 271), P271 lost CH_2_O to generate P241 (C_14_H_13_N_2_O_2_^+^, *m/z* = 241), and finally, P241 lost C_2_H_4_O_2_ to generate P181 (C_12_H_9_N_2_^+^, *m/z* = 181)^[46]^. P329 was present as an isomeride that was named P329-2 (*m/z* = 329). The intermediate products, P329-2, P301 (*m/z* = 301), P200 (*m/z* = 200) and P158 (*m/z* = 158), are shown in Table S7, and their possible degradation pathways are shown in Fig. S4b. P329-2 first lost CO to generate P301 (C_16_H_17_N_2_O_4_^+^, *m/z* = 301), P301 lost C_4_H_7_NO_2_ to generate P200 (C_12_H_10_NO_2_^+^, *m/z* = 200), and finally, P200 lost C_2_H_2_O to generate P158 (C_10_H_8_NO^+^, *m/z* = 158)^[46]^.

***Question 24*** *Results and discussion:*

*- Author should add text to explain why “the degradation kinetics of NIF via UV/H_2_O_2_ system was linear decrease with SO_4_^2-^ concentration increasing.” “the degradation kinetics of NIF via UV/H_2_O_2_ system was linear decrease with CO_3_^2-^ concentration increasing” … instead of provide equations E3- E41.*

**Answer.**

The degradation rate of NIF decreased with increasing divalent anions (SO_4_^2-^ and CO_3_^2-^) was due to the quenching effect. See the answers of ***Question 21***.

***Question 25*** *Results and discussion:*

*- Author should add text to explain why “the degradation kinetics of NIF via UV/H_2_O_2_ system was linear decrease with SO_4_^2-^ concentration increasing.” “the degradation kinetics of NIF via UV/H_2_O_2_ system was linear decrease with CO_3_^2-^ concentration increasing” … instead of provide equations E3- E41.*

**Answer.**

See the answers of ***Question 21***.

**Response to 4^th^ reviewer**

***Question 26*** *The whole article has serious errors with English and the sentence drafting, it is highly urged to fix it.*

**Answer.**

The language of manuscript (grammar, punctuation, spelling, and overall style) was corrected by the *Nature Research Editing Service* (website: <https://secure.authorservices.springernature.com/>). The “**Editing Certificate**” with a verification code [0C36-F874-B639-2E32-1B7F] was used to verify by secure.authorservices.springernature.com/certificate/verify. The detailed changes of this manuscript were in “**Manuscript**”, “**Revised Manuscript with Track Changes**” and “**Supporting Information**”.

***Question 27*** *Authors have used EPR studies, more experimental conditions like magnetic field, temperature etc need to be specified. This are very important parameters.*

**Answer.**

The ESR measurements was in [**2.5 Electron spin resonance (ESR) measurements**] and [**3.7 Oxidation mechanism and degradation pathway of NIF *via* UV/H_2_O_2_**].

For **[2.5** **Electron spin resonance (ESR) measurements]**

ESR measurements were performed with a JES-FA200 electron spin resonance spectrometer and used to measure the hydroxide radical (·OH) and superoxide radical (·O_2_^-^) during the degradation of NIF under UV/H_2_O_2_ using 5,5-dimethyl-1-pyrroline N-oxide (DMPO) as the spin trapping reagent.

For **[3.7 Oxidation mechanism and degradation pathway of NIF *via* UV/H_2_O_2_]**

**3.7 Oxidation mechanism and degradation pathway of NIF *via* UV/H_2_O_2_**

In recent advances in UV/H_2_O_2_ systems, the degradation of organic pollutants has been due to the generation of ROS, especially ·OH and ·O_2_^-^. The oxidation mechanism of NIF degradation *via* the UV/H_2_O_2_ system was measured by ESR measurements, and the ESR spectra are shown in Fig. 9(a-b). The significant ·OH signal (Fig. 9a) showed four peaks at 321.8 mT (P_A_), 323.3 mT (P_B_), 324.8 mT (P_C_) and 326.3 mT (P_D_). The interspaces of P_A_-P_B_, P_B_-P_C_ and P_C_-P_D_ were constant of 1.5 mT, and the intensity ratio of P_A_, P_B_, P_C_ and P_D_ was 1:2:2:1^[41]^. The significant ·O_2_^-^ signal (Fig. 9b) showed four peaks at 322.1 mT (P’_A_), 323.2 mT (P’_B_), 324.4 mT (P’_C_) and 325.8 mT (P_D_). The interspaces of P’_A_-P’_B_, P’_B_-P’_C_ and P’_C_-P’_D_ were constant within the range of 1.0 mT from 1.5 mT, and the intensity ratio of P’_A_, P’_B_, P’_C_ and P’_D_ was 1:1:1:1^[42]^. However, the intensity of the ·OH signal was stronger than that of the ·O_2_^-^ signal, which means that ·OH was the primary key ROS and ·O_2_^-^ was the secondary key ROS. The oxidation mechanism of NIF degradation *via* the UV/H_2_O_2_ system is shown in Equations 33 (E33) to 41 (E41)^[43]^. The generation of ·OH mainly comes from the direct decomposition of H_2_O_2_ (E33), the generation of ·O_2_^-^ mainly comes from the indirect reaction between oxygen gas (dissolved oxygen and H_2_O_2_ decomposition) and electrons (E35 and E38), and NIF is degraded *via* the ROS (E41)^[44]^.

H_2_O_2_ + *hv* → 2 ·OH	(E33)

H_2_O_2_ + ·OH → ·HO_2_ + H_2_O	(E34)

·HO_2_ → ·O_2_^-^ + H^+^	(E35)

2 ·HO_2_ → H_2_O_2_ + O_2_	(E36)

·HO_2_ + ·OH → H_2_O + O_2_	(E37)

O_2_ + e^-^ → ·O_2_^-^	(E38)

2 ·O_2_^-^ → ^1^O_2_ + ^3^O_2_ + 2 e^-^	(E39)

2 ·OH → H_2_O_2_	(E40)

NIF + ROS → Intermediate Products → CO_2_ + H_2_O	(E41)

**Fig. 9 ESR measurements of ·OH (a) and ·O_2_^-^ (b).**

***Question 28*** *Authors have repeated the data shown in different table in the manuscript as well while explanation, I think this is not comfortable and does not make any sense for the repetition of data.* *This needs to be removed. Authors should use values from different tables to explain their observations and restricts themselves by writing all the number in manuscript.*

**Answer.**

The Table 1, Table 2, Table 3, Table 4, Table 5 and Table 6 were deleted in Manuscript. And the Table 1, Table 2, Table 3, Table 4, Table 5 and Table 6 were changed to Table S1, Table S2, Table S3, Table S4, Table S5 and Table S6 in Supporting Information to help readers understand the degradation performance of NIF *via* UV/H_2_O_2_ system.

***Question 29*** *Authors should perform a study on the effect of NIF removal for different initial concentration of the NIF.*

**Answer.**

The removal of NIF via UV/H_2_O_2_ was fast. When the NIF concentration was low, it is difficult to test degradation performance in less than 1 min; however, when the NIF concentration was low, it is not existing in the real water environment. Hence, after many experiments, the NIF concentration = 5 mg/L was selected in this study.

***Question 30*** *Authors have mentioned that pH of the water shows linear decrease with increase in pH value, however as far as I can see the from 4-7 the removal rate is almost constant. How to justify this?*

**Answer.**

The effect of pH was shown in [**3.2 Effect of initial pH**] as following:

For [**3.2 Effect of initial pH**]

The pH is another key parameter of the UV/H_2_O_2_ system. It significantly affects the oxidative degradation of antibiotics by transforming the protonation states and changing the redox potential at different pH values. As shown in Fig. 4(a-b) and Table S2, the degradation rates of NIF under UV/H_2_O_2_ was 97.54% (pH = 10) < 98.69% (pH = 8) < 99.77% (pH = 5) < 99.94% (pH = 4 and 7), the kinetics constant k’_app_ was 0.75217 min^-1^ (pH = 10) < 0.91269 min^-1^ (pH = 8) < 1.21831 min^-1^ (pH = 5) < 1.45569 min^-1^ (pH =7) < 1.51175 min^-1^ (pH = 4), and the t_1/2_ was 0.4 min (pH =7) < 0.7 min (pH = 4) < 0.8 min (pH = 5) < 0.9 min (pH = 8) < 1.1 min (pH = 10) when the NIF concentration = 5 mg/L, pH = 4-10, H_2_O_2_ dose = 0.52 mmol/L, T = 20 ℃ and reaction time = 5 min. As shown in Fig. 4c, the degradation kinetics of NIF *via* UV/H_2_O_2_ system exhibited a poor linear decrease as the pH increased (y = -0.1155x + 1.95533, R^2^ = 0.6884). The results indicated that acidic solution (pH = 4) was more favorable for degrading NIF than basic solution (pH = 10) under UV/H_2_O_2_. The possible reasons were in accord with the redox potential, generation rate of ROS and reaction rate between ROS and NIF. The inhibiting effect of the basic solution was due to the quenching reaction between OH^-^ and ·OH, which is shown in Equations 7 (E7) to 11 (E11)^[36]^:

H_2_O_2_ → HO_2_^-^ + H^+^	(E7)

H^+^ + OH^-^ → H_2_O	(E8)

H_2_O_2_ + HO_2_^-^ → H_2_O + OH^-^ + O_2_	(E9)

·OH + HO_2_^-^ → OH^-^ + ·HO_2_	(E10)

·OH + HO_2_^-^ → H_2_O + ·O_2_^-^	(E11)

**Fig. 4 Effect of initial pH on the removal rate (a), kinetics constant (b) and linear fitting between kinetics constant and initial pH on the degradation of NIF *via* UV/H_2_O_2_. The error bars represent the standard deviation (n = 3).**
