# Supplementary material for: Highly efficient UV/H2O2 technology for the removal of nifedipine antibiotics: Kinetics, co-existing anions and degradation pathways
Source: PLoS One. 2021 Oct 28;16(10):e0258483. doi: 10.1371/journal.pone.0258483 (PMC8553136; doi:10.1371/journal.pone.0258483)
Supplement: Attachment — Submitted filename: Response to Reviewers.doc [file pone.0258483.s016.doc]

**Response to Editor's comments:**

Thank you very much for your consideration of our manuscript entitled “**Highly efficient UV/H_2_O_2_ technology for the removal of nifedipine antibiotics: Kinetics, co-existing anions and degradation pathways**” (PONE-D-21-20580R1) to ***Plos One***.

We have read the editor’s comments and reviewers’ questions carefully, and made an attempt to respond as clearly as possible. In reference to the above recommendations, the responses are shown in “Response to 3^rd^ reviewer”.

**Response to Journal Requirements:**

*Please review your reference list to ensure that it is complete and correct. If you have cited papers that have been retracted, please include the rationale for doing so in the manuscript text, or remove these references and replace them with relevant current references. Any changes to the reference list should be mentioned in the rebuttal letter that accompanies your revised manuscript. If you need to cite a retracted article, indicate the article’s retracted status in the References list and also include a citation and full reference for the retraction notice.*

**Answer.**

The references were checked without any questions.

The references [15] and [16] were not the retraction paper.

The authors, title, journal, year and doi of references [15] and [16] were correct and searchable although without issue, volume and pages.

[15] Gunarathne V, Rajapaksha AU, Vithanage M, Alessi DS, Selvasembian R, Naushad M, You SM, Oleszczuk P, Ok YS. Hydrometallurgical processes for heavy metals recovery from industrial sludges. Crit Rev Env Sci Tec. 2020; 10.1080/10643389.2020.1847949.

[16] Hariharan A, Harini1 V, Sandhya S, Rangabhashiyam S. Waste *Musa acuminata* residue as a potential biosorbent for the removal of hexavalent chromium from synthetic wastewater. Biomass Convers Bior. 2020; 10.1007/s13399-020-01173-3.

**Response to 3^rd^ reviewer:**

***Question 12:*** *The novel of study should be mentioned clearly. The combination of UV light with hydrogen peroxide (UV/H_2_O_2_) to degrade organic pollutants is not new, so the author should reveal what difference is?*

**Answer.**

Thanks a lot. The novelty of this paper was mentioned in **[Introduction]** as following:

A novel method of UV light combined with hydrogen peroxide (UV/H_2_O_2_) is highly efficient, fast, and has a strong oxidizing ability; these advantages are attributed to the synergistic ability of UV light and H_2_O_2_ to generate ROS^[30]^. However, in UV/H_2_O_2_ AOPs, other constituents in water matrices may significantly affect the removal of target contaminants by competitively interacting with photons and ROS. In our previous study, the degradation of norfloxacin by using UV/H_2_O_2_ was investigated^[31]^. The degradation rate and apparent first-order kinetics constant of norfloxacin *via* UV/H_2_O_2_ were 98.8% and 0.22248 min^-1^, respectively, and the norfloxacin concentration = 20 mg/L, the H_2_O_2_ dose =1.2 mmol/L, the pH=7, T= 20 °C and the reaction time = 20 min. The kinetics were low, and the formation mechanism of ROS was controversial, but it provided a novel research direction for the degradation of NIF *via* a UV/H_2_O_2_ system. Therefore, it should be noted that the degree of research to date on the degradation of NIF *via* UV/H_2_O_2_ oxidation processes is insufficient to thoroughly understand the fundamentals of ·OH generation, intermediate products and degradation pathways, which are important processes that must be considered in the design of wastewater treatment technology^[32]^. Furthermore, the effect of co-existing anions in the UV/H_2_O_2_ system may significantly affect the removal of NIF by competitively quenching with ROS^[33]^. Thus, it is still challenging to design a UV/H_2_O_2_ wastewater treatment technology with high efficiency.

On the one hand, the oxidizability of UV/H_2_O_2_ AOPs and removal rate of NIF were enhanced due to the combination between UV and H_2_O_2_^[30]^. On the other hand, the anion (such as NO_3_^-^) was generated due to the degradation reaction between NIF and ROS. However, impacts of NO_3_^-^ showed duality: it promotes the generation of ROS under irradiation, and also quenches the ROS of UV/H_2_O_2_^[32-33]^. Hence, it is significant and meaningful to study the effect of co-existing anions on the degradation of NIF via UV/H_2_O_2_ AOPs. To better understand the removal efficacy of a target compound by UV/H_2_O_2_ AOPs in different real water environments, the divalent anions (SO_4_^2-^ and CO_3_^2-^) and monovalent anions (Cl^-^ and NO_3_^-^) have been developed to model the impact of water constituents on the reaction kinetics.
